# Supplementary material for: Novel model to predict risk of invasive fungal infection and fungal prophylaxis timing
Source: Microbiol Spectr. 2025 Oct 13;13(11):e02958-24. doi: 10.1128/spectrum.02958-24 (PMC12584638; doi:10.1128/spectrum.02958-24)

**Trail Protocol**

Novel clinical model to predict risk of invasive fungal infection and fungal prophylaxis timing determination

Address: Number 221, West Yan’ an Road

Phone: 62483180

Postal Code: 200040

PI: Jiexian Ma

Email: majiexian@fudan.edu.cn

**Background：**

Invasive fungal infection (IFI) is the leading cause of death in patients with hematological malignancies, especially those who have received strong cytotoxic chemotherapy, immunosuppressants, or hematopoietic stem cell transplantation (HSCT) [1,2]. Invasive fungal infections have been increasing year by year in the past few decades, mainly related to the emergence of HSCT and the increased use of chemotherapy and immunosuppressants in patients with hematological diseases [3,4]. The analysis of the Transplant Associated Infection Monitoring Network (TRANSNET) database shows that the cumulative annual invasive fungal infection rates per 100 transplants for patients undergoing fully matched allogeneic transplantation, non-identical allogeneic transplantation, and autologous HSCT are 7.7, 8.1, and 1.7, respectively [2]. Long term use of glucocorticoids, immunosuppressants, and radiotherapy and chemotherapy also increase the incidence of invasive fungal infections in patients. The prognosis of invasive fungal infections in patients with hematological diseases is poor. The reported mortality rate of IFI ranges from 50% in leukemia or lymphoma patients to 87% in hematopoietic stem cell transplant patients [5]. In the TRANSNET database, the 1-year survival rates for invasive candidiasis and invasive aspergillosis are 33% and 25%, respectively [3]. Due to its high mortality rate, prevention of invasive fungal infections is extremely important. However, excessive fungal prophylaxis also has related side effects and can lead to drug resistance [6]. Currently, guidelines only [7] stipulate that patients with grade III to IV graft-versus-host disease after chemotherapy for acute myeloid leukemia and allogeneic hematopoietic stem cell transplantation should undergo fungal prophylaxis. In clinical practice, it has been found that invasive fungal infections are far from sufficient. Many critically ill patients, such as those in the ICU who have been using large amounts of glucocorticoids and immunosuppressants for a long time, as well as those who have undergone organ transplantation and hematopoietic stem cell transplantation, still frequently experience invasive fungal infections. Identifying the timing of starting and stopping fungal prevention in critically ill patients remains a challenging task. However, there is currently no consensus in this field. Most clinical doctors rely on experience without measurable indicators, and there are still many issues worth exploring.

It is generally believed that the following critically ill patients are high-risk groups for invasive fungal infections: ① those who have received or are currently receiving immunosuppressive therapy or radiation therapy within the previous 30 days (oral immunosuppressive therapy>2 weeks or intravenous chemotherapy>2 courses); ② Long term use of glucocorticoids (intravenous or oral equivalent to prednisone 0.5mg/kg/d for more than 2 weeks); ③ Organ transplant patients; ④ Patients with diabetes, COPD and tumor. In the preliminary work of our center, we retrospectively collected 141 critically ill patients who had long-term use of large amounts of immunosuppressants or received autologous or allogeneic hematopoietic stem cell transplantation for hematological diseases but had recovered from granulocyte deficiency. According to the guidelines, these patients do not need IFI prevention. During follow-up, we found that 25 patients developed invasive fungal infections. We retrospectively analyzed the cellular and humoral immune function of 141 patients with allogeneic or autologous HSCT, including peripheral blood immunoglobulin levels, CD4+, CD8+, NK cells, B lymphocyte counts, CD4/CD8 cell ratios, and analyzed the relationship between IFI incidence and these indicators. As a result, it was found that only the decrease in IgG levels in the body and the decrease in NK cells were associated with invasive fungal infections. In the later stage, logistic regression analysis and ROC curve were used to explore the relationship between immune function and IFI. It was found that fungal prevention should be started when IgG was below 7mg/ml and NK cell count was below 6.5 * 104/L, and stopped when the count recovered. This study has been published in the internationally influential SCI journal Frontiers in Microbiology (IF=4.10) [8]. Another study on immune function monitoring in patients after liver transplantation also showed that the CD4/CD8 cell ratio was not associated with invasive fungal infections, which is consistent with our research [9].

We subsequently enrolled nearly 1000 patients, and by comparing the relationship between their immune function and fungal infections, we found that Multivariate logistic regression analysis further validated independent risk factors for IFI with age, IgG level below 6.5 g/L, and CD4+ T cell count retaining significance with incidence of IFI, The three independent prognostic factors (age, IgG level, and CD4+ T cell count) were incorporated into a predictive model to estimate the probability of invasive fungal infection ,Patients with a total score above 115 points were classified as high-risk IFI patients：


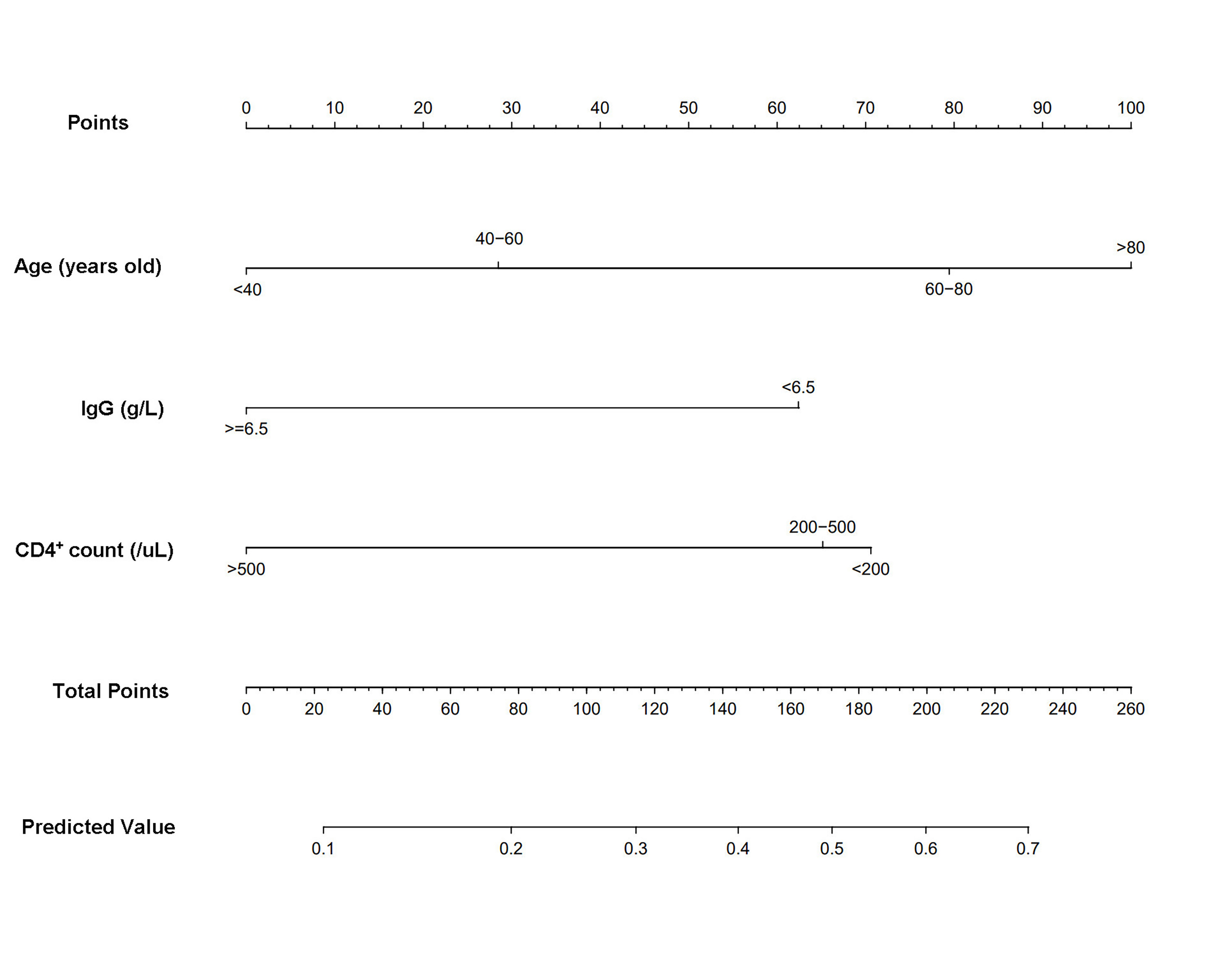


1. Patients who undergo autologous hematopoietic stem cell transplantation and allogeneic hematopoietic stem cell transplantation, have received strong cytotoxic drugs (such as high-dose CTX, cladribin), high-dose long course corticosteroids (0.5mg/kg prednisone for more than 1 month), high-dose long course immunosuppressants (for more than 1 month), or have used drugs such as rituximab, do not require fungal prophylaxis according to current guidelines (guidelines only stipulate that patients who have experienced grade III to IV graft-versus-host disease during allogeneic hematopoietic stem cell transplantation need fungal prophylaxis).
2. Monitor immunoglobulin levels and cellular immunity (T cell, B cell, NK cell count) monthly.

③According to the scoring in the table above, each patient will be divided into a high-risk group and a low-risk group based on their scores. The high-risk group will be randomly divided into two groups using a random table: the fluconazole prevention group and the non-prevention group. The endpoint of the study is the occurrence of invasive fungal infection in patients or the recovery of their immune function.

④Compare the differences in the incidence of invasive fungal infections between the fungal prevention and non-prevention groups and the low-risk group based on immune monitoring, as well as the economic benefits of prevention and non-prevention.

This study starts from clinical practical problems and relies on the results of previous retrospective studies. Through prospective cohort studies, it elucidates the relationship between fungal prevention and immune function in critically ill patients, and further clarifies the monitoring indicators, timing, and drugs used to initiate and terminate fungal prevention. If this work is successful, it can further improve fungal prevention guidelines and guide clinical work, which may greatly reduce the incidence of invasive fungal infections, benefit patients, and save a lot of medical resources. It has great social and economic value.

**Reference：**

1. Lin, S. J., Schranz, J., and Teutsch, S. M. (2001). Aspergillosis case-fatality rate: systematic review of the literature. Clin. Infect. Dis. 32, 358–366. doi: 10.1086/ 318483
2. Gavalda, J., Len, O., San Juan, R., Aguado, J. M., Fortun, J., Lumbreras, C., et al. (2005). Risk factors for invasive aspergillosis in solid-organ transplant recipients:a case-control study. Clin.Infect.Dis. 41,52–59. doi:10.1086/43060
3. Kontoyiannis, D. P., Marr, K. A., Park, B. J., Alexander, B. D., Anaissie, E. J., Walsh,T.J.,et al.(2010).Prospective surveillance for invasive fungal infections in hematopoietic stem cell transplant recipients, 2001–2006: overview of the Transplant-Associated Infection Surveillance Network (TRANSNET) Database. Clin.Infect.Dis. 50,1091–1100. doi:10.1086/651263
4. Pagano, L., Caira, M., Candoni, A., Oﬃdani, M., Martino, B., and Specchia, G. (2010). Invasive aspergillosis in patients with acute myeloid leukemia: a SEIFEM-2008 registry study. Haematologica 95, 644–650. doi: 10.3324/ haematol.2009.012054
5. Kurosawa, M., Yonezumi, M., Hashino, S., Tanaka, J., Nishio, M., Kaneda, M., et al.(2012).Epidemiology and treatment outcome of invasive fungal infections in patients with hematological malignancies. Int. J. Hematol. 96, 748–757. doi:10.1007/s12185-012-1210-y
6. Martín-Peña,A.,Aguilar-Guisado,M.,andCisneros,J.M.(2014).Doesthecurrent treatment of invasive fungal infection need to be reviewed? Enferm. Infecc. Microbiol.Clin.32,523–528.doi:10.1016/j.eimc.
7. ChineseInvasiveFungalInfectionWorkingGroup(2017).TheChineseguidelines for the diagnosis and treatment of invasive fungal disease in patients with hematological disorders and cancers (the ﬁfth revision). Zhonghua Nei Ke Za Zhi56,453–459.doi:10.3760/cma.j.issn.0578-1426.2017.06.015

8. [Jiexian Ma](https://pubmed.ncbi.nlm.nih.gov/?term=Ma+J&cauthor_id=29552004) , [Yingwei Hu](https://pubmed.ncbi.nlm.nih.gov/?term=Hu+Y&cauthor_id=29552004) , [Min Wu](https://pubmed.ncbi.nlm.nih.gov/?term=Wu+M&cauthor_id=29552004) , [Xiaoqin Wang](https://pubmed.ncbi.nlm.nih.gov/?term=Wang+X&cauthor_id=29552004) , [Yanhui Xie](https://pubmed.ncbi.nlm.nih.gov/?term=Xie+Y&cauthor_id=29552004) . Timing Determination of Invasive Fungal Infection Prophylaxis According to Immune Function in HSCT Patients. Front Microbiol. 2018 Mar 2;9:370.

9. [Tao Zhou](https://pubmed.ncbi.nlm.nih.gov/?term=Zhou+T&cauthor_id=22118697), [Feng Xue](https://pubmed.ncbi.nlm.nih.gov/?term=Xue+F&cauthor_id=22118697), [Long Zhi Han](https://pubmed.ncbi.nlm.nih.gov/?term=Han+LZ&cauthor_id=22118697), [Zhi Feng Xi](https://pubmed.ncbi.nlm.nih.gov/?term=Xi+ZF&cauthor_id=22118697), [Qi Gen Li](https://pubmed.ncbi.nlm.nih.gov/?term=Li+QG&cauthor_id=22118697), [Ning Xu](https://pubmed.ncbi.nlm.nih.gov/?term=Xu+N&cauthor_id=22118697), et al. Invasive fungal infection after liver transplantation: risk factors and significance of immune cell function monitoring. J Dig Dis. 2011 Dec;12(6):467-75.

**Research objective:**

1) Clarify the relationship between cellular and humoral immune functions and invasive fungal infections.

2) Supplementary guidelines on the economic benefits of fungal prophylaxis for severe immunodeficiency patients based on immune function monitoring.

**Types of experimental designs, randomization methods, and levels of blinding**

1. Referring to the results of previous retrospective studies, the sample size was calculated (alpha=0.05, bata=0.1，OR=10）， The sample size for each group is calculated to be only 30 cases. Considering indicators such as leakage, each group will have 40 enrolled patients. Therefore, the minimum number of enrolled patients in this study is about 120. The main endpoint of the study is the occurrence of invasive fungal infections, and the secondary endpoint is the complete recovery of patient immune monitoring to normal or patient death.
2. **Inclusion Criteria**，Screening patients: The enrolled patients include the following characteristics:
3. Autologous hematopoietic stem cell transplantation and allogeneic hematopoietic stem cell transplantation, or ② patients who have received strong cytotoxic drugs (such as high-dose CTX) or ③ patients who have received high-dose long-term corticosteroids (0.5mg/kg prednisone for more than 1 month), or ④ patients who have used immunosuppressive drugs such as rituximab or targeted drugs that may affect immunity, and who have recovered from granulocyte deficiency or have no granulocyte deficiency, and do not require fungal prophylaxis according to current guidelines (i.e. patients who have experienced grade III to IV graft-versus-host disease during allogeneic hematopoietic stem cell transplantation are excluded).

**The exclusion criteria** are: ① Patients with acute myeloid leukemia or other hematological malignancies who have undergone chemotherapy and have a granulocyte deficiency phase exceeding one week. ② Patients undergoing allogeneic hematopoietic stem cell transplantation who have experienced grade III to IV acute graft-versus-host disease and require fungal prophylaxis according to guidelines

③ Participants who have participated in other clinical trials within 30 days;

④Pregnant and lactating patients;

⑤The patient has other types of tumors, but excluding superficial bladder cancer, basal cell or squamous cell carcinoma of the skin, cervical intraepithelial neoplasia (CIN) or prostatic intraepithelial neoplasia (PIN);

⑥ Active viral or bacterial infections that have not been controlled with appropriate anti-infective treatments;

⑦The serological reaction of known HIV or active hepatitis C virus is positive; Active hepatitis B virus is characterized by increased HBV titer and abnormal liver function. Such patients need strict anti-hepatitis B virus treatment, and can be considered into the group only after hepatitis B virus is controlled (HBV titer drops). Patients with big three positive and small three positive hepatitis B, such as HBV DNA<500, can be enrolled after regular antiviral treatment.

8.Patients with mental illnesses or other conditions who are unable to cooperate with research, treatment, and monitoring requirements;

3）Monthly monitoring of immunoglobulin levels (including IgG levels, IgA levels, IgE levels, IgM levels) and cellular immunity (T cell, B cell, NK cell numbers, CD4, CD8, CD19, CD56+cell percentages) is conducted on these patients. Based on the scores, those with scores above 115 are identified as high-risk groups.

4) For high-risk patients, they were randomly divided into two groups based on a randomization scale, one receiving fluconazole prophylaxis and the other receiving no prophylaxis.

5) The dosage of fungal prophylactic drugs in the fluconazole group is fluconazole 200mg, twice a day; The endpoint of the study is the recovery of the patient's immune function, i.e. IgG above 7mg/ml and CD4+T cell count above 200/uL or the occurrence of invasive fungal infection in the patient. The diagnostic criteria for invasive fungal infection include confirmed diagnosis and clinical diagnosis. Clinical diagnosis includes clear pathogenic evidence, while clinical diagnosis includes high-risk factors for invasive fungal disease. There is no clear pathogenic evidence, but the G test or GM test is positive. The endpoint of the study is the recovery of the patient's immune function, i.e. IgG above 7mg/ml and NK cell count above 6.5-104/L or the occurrence of invasive fungal infection in the patient. And it is necessary to record the patient's hospitalization expenses for the treatment of fungal infections.

6) Compare the differences in the incidence of invasive fungal infections between fungal prevention and non-prevention based on immune monitoring, as well as the economic benefits of using fluconazole for prevention and non-prevention.

**Technical route:**

**The research technology route is as follows:**


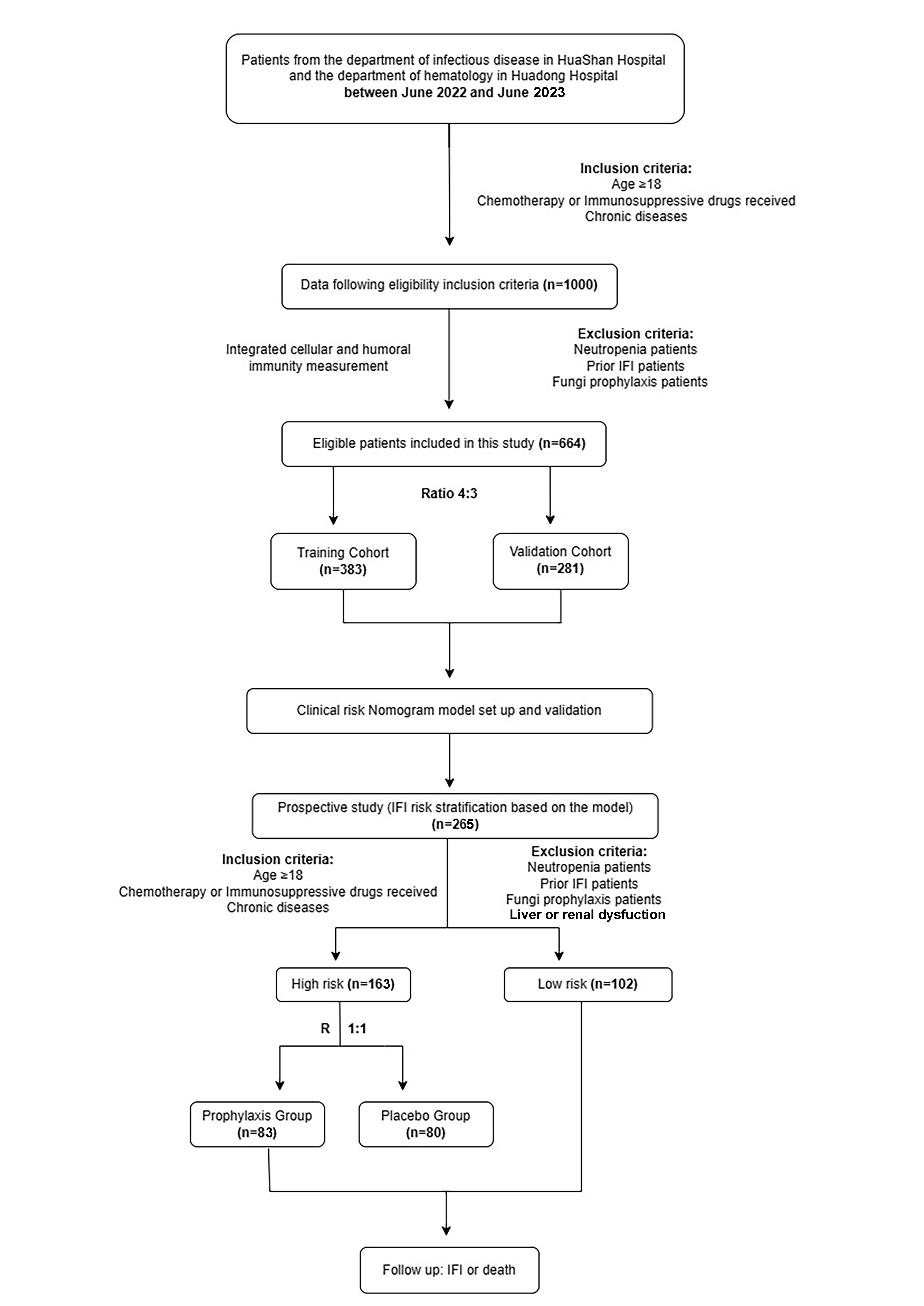

Supplement: Data S2 — Trial protocol. [file spectrum.02958-24-s0002.doc]
