# Supplementary material for: Novel model to predict risk of invasive fungal infection and fungal prophylaxis timing
Source: Microbiol Spectr. 2025 Oct 13;13(11):e02958-24. doi: 10.1128/spectrum.02958-24 (PMC12584638; doi:10.1128/spectrum.02958-24)

Supplemental materials

Table S1. Antifungal Regimens for Suspected/Proven Invasive Fungal Infection

Antifungal Regimens	Low-risk Patients (N=102)	High-risk Patients (N=163)		p=0.850
		Non-fungal Prophylaxis (N=80)	Fungal Prophylaxis (N=83)	
**Voriconazole**	**7**	**18**	**5**	
**Caspofungin**	**5**	**17**	**4**	
**Amphotericin B liposomal**	**0**	**2**	**0**	

Figure S1. Calibration plots

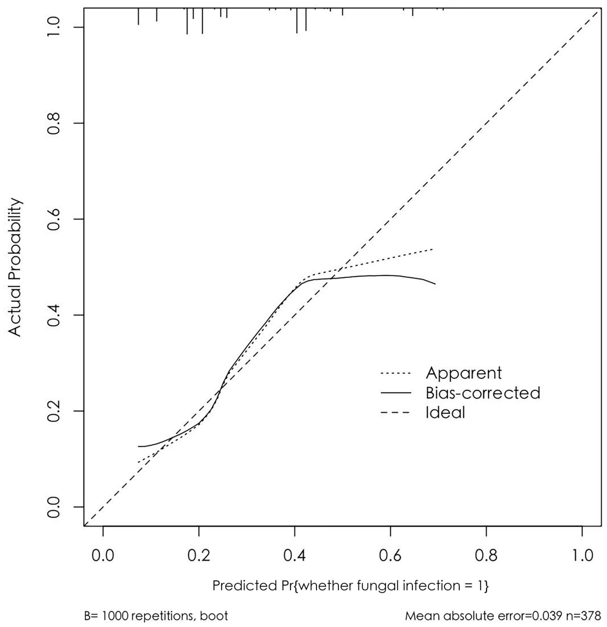

Supplement: Supplemental material — Fig. S1; Table S1. [file spectrum.02958-24-s0003.pdf]
